# The Prevalence, Risk Factors, and Clinical Correlates of Erosive Esophagitis and Barrett's Esophagus in Iranian Patients with Reflux Symptoms

**DOI:** 10.1155/2014/696294

**Published:** 2014-03-17

**Authors:** Alireza Sharifi, Shahab Dowlatshahi, Hedieh Moradi Tabriz, Fatemeh Salamat, Omid Sanaei

**Affiliations:** ^1^Division of Gastroenterology, Department of Medicine, Sina Hospital, Tehran University of Medical Sciences, Tehran 1136746911, Iran; ^2^Department of Pathology, Sina Hospital, Tehran University of Medical Sciences, Tehran 1136746911, Iran; ^3^Chancellorship for Research, Guilan University of Medical Sciences, Rasht 4193893345, Iran; ^4^Golestan Research Center of Gastroenterology and Hepatology, Golestan University of Medical Sciences, 10th Azar Alley, 5 Azar Boulevard, Gorgan 4917774979, Iran

## Abstract

*Background.* Erosive esophagitis (EE) and Barrett's esophagus (BE) are the two important complications of gastroesophageal reflux disease. We aimed to investigate the prevalence of and the risk factors for EE and BE in an Iranian group of patients with reflux symptoms. We also examined the relationship between reflux symptoms and endoscopic findings. *Methods.* A total of 736 patients with gastroesophageal reflux disease (GERD) symptoms were enrolled and all underwent upper gastrointestinal endoscopy. Diagnosis of Barrett's esophagus was confirmed by pathologic examination and Helicobacter pylori (H. pylori) infection was demonstrated by rapid urease test. *Results.* Two hundred eighty-three and 34 patients were found to have EE and BE, respectively. Multivariate analysis showed that hiatal hernia (*P* < 0.001) and H. pylori infection (*P* < 0.002) were the two significantly related risk factors for esophagitis. Only age was related to BE, with BE patients being more likely to be older (*P* < 0.001) than others. * Conclusions.* Prevalence of EE and BE in Iranian reflux patients is similar to that seen in western countries. H. pylori infection and the presence of hiatal hernia may be strong risk factors for esophagitis as does older age for Barrett's esophagus. Finally, reflux symptoms have no significant relationship with endoscopic findings.

## 1. Introduction

GERD is a prevalent condition in western countries, affecting about 10% to 20% of general population [1]. Barrett's esophagus (BE) and erosive esophagitis (EE) are the two commonly seen complications of GERD. Different risk factors have been proposed for GERD in eastern and western countries, but the data regarding how these factors contribute to the development of GERD complications are inconsistent. Since GERD is a fairly common problem in Iran [2], the disorder and its complications need to be addressed carefully. To do this, in the present study we decided to investigate the prevalence of BE and EE in a group of patients undergoing upper GI endoscopy for reflux symptoms. We then studied whether gender and Helicobacter pylori (H. pylori) infection have any contribution to the development of BE and EE.

It has been shown that 10% to 70% of patients with GERD may have relevant endoscopic findings, whereas esophagitis evidence may present in about 8% of asymptomatic individuals [3]. Similarly, BE does not develop in all patients with EE who have a history of gastroesophageal reflux (GER) [4], while up to 25% of patients with BE may not have any GERD history [5]. As a result, we also investigated the relationship between clinical symptoms of GERD patients with their endoscopic findings. In this regard, we evaluated the clinical correlates of GERD patients whose esophagitis had been graded according to the Los Angeles (LA) classification [6].

## 2. Materials and Methods

The study was conducted in Sina hospital, a general medical and surgical university hospital in the metropolitan city of Tehran (the capital of Iran with an estimated population of 15 million), which provides state-of-the-art care for millions of Tehranian population. We enrolled 736 consecutive patients with GERD symptoms who presented at the gastroenterology outpatient clinic of Sina hospital; none of them had been referred by a general practitioner or a specialist. In this study we defined GERD as a condition where patients must have mild reflux symptoms in two or more days per week or moderate to severe symptoms at least in one day per week [7]. Severity of heartburn was assessed by a gastroenterologist expert in GERD during a clinical visit. Our criteria for heartburn severity were as follows: mild, awareness of symptom but easily tolerated; moderate, discomfort sufficient to cause interference with normal activities; severe, incapacitating, with inability to perform normal activities [8]. Patients with a history of documented peptic disease, gastric or esophageal surgery and those with motor disorders such as achalasia, diffuse esophageal spasm, or scleroderma were excluded.

The study protocol was approved by local ethics committee and all patients gave their written informed consent to be included. All patients were informed completely of the study protocol and consented to undergo upper GI endoscopy.

All upper endoscopies were performed using a GIF100 or GIF130 video endoscope (Olympus, Lake Success, NY). EE was defined as the presence of one or more mucosal injury at the distal esophagus on endoscopy (erosion or ulceration). The diagnosis of hiatal hernia was confirmed by the presence of gastric folds ≥3 cm above the diaphragmatic pinch. Gastric and duodenal ulcers were also defined as lesions at least 0.5 cm in diameter, possessing unequivocal depth, and located in any portion of the stomach or duodenal bulb. Based on the endoscopic findings, subjects were divided into three groups including patients with Normal-appearing Esophagogastric Junction (NEJ), those with EE and those who had Barrett's esophagus. Of note, patients with coexisting Barrett's esophagus and esophagitis were included in the Barrett's group when comparing BE patients with others and in the esophagitis group when comparing EE with NEJ.

Suspected BE was biopsied from four quadrants of the involved epithelium, 2 cm apart. All biopsy specimens were stained with hematoxylin and eosin (H&E) and with alcian blue (pH 2.5) stain. Specimens were then examined by two expert pathologists for the presence of intestinal metaplasia defined by the presence of clear goblet cells within the columnar epithelium. By using pathology and rapid urease test (RUT), the presence of H. pylori infection was also investigated in specimens obtained from antrum.

## 3. Statistical Analysis

Descriptive analysis was done and frequencies and percentages of the categorical variables were calculated. To summarize quantitative variables we used means and standard deviations.

Where needed, the Fisher's exact test and the two-sample *t*-test were used to test the association of esophageal lesions (BE and/or EE) with the patient demographics, the presence of hiatal hernia and a positive RUT. Thereafter, logistic regression was performed to identify variables predictive of BE or EE. These variables included gender, age, BMI, cigarette smoking, alcohol use, H. pylori infection and size of the hiatal hernia. To estimate the adjusted odds ratio (OR) for each variable, we included variables with a *P* < 0.25, obtained from univariate analysis, as independent factors in the forward stepwise logistic regression. Finally, multivariate analysis was done to find factors which were significantly related to the development of EE or BE. A two-tailed *P* < 0.05 was considered statistically significant.

## 4. Results

A total of 736 patients with a mean age of 48.9 years (median: 50, range: 15–86) were included. Four hundred eleven (55.8%) were male and 325 (44.2%) were female. EE was found in 283 patients (38.5%), and was graded according to LA classification. Of them, 218 (77%) patients were in LA class A, 51 (18%) in B, 9 (3.2%) in C and 5 (1.8%) in class D. Only 34 patients (4.6%) had Barrett's esophagus, 14 of them had concomitant EE with 11 being in LA class A, 2 in B and 1 in class D. No esophageal stricture or adenocarcinoma was found.

Eighty eight (12%) patients had endoscopic evidence of peptic ulcer disease. Of these, 28 had gastric ulcer (3.8%) and 60 had duodenal ulcer (8.2%). Notably, in 51 of these patients there was not any endoscopic evidence of EE. Hiatal hernia was detected in 106 patients (14.4%). Finally, RUT demonstrated H. pylori infection in 216 individuals.

Demographic characteristics of the patients with EE and NEJ are shown in Table 1. Both groups were comparable in terms of sex (*P* = 0.30) and age (*P* = 0.08). NEJ patients included 235 men (54.3%) and 198 women (45.7%) ranging in age from 16 to 85 years (mean 48 ± 16.6, median = 49). The mean age of patients with esophagitis was 50.14 years and 58% of them were male. In the same way, BE patients had a mean age of 59.19 years with 67.6% of them being male. Notably, the proportion of men in patients with BE was higher compared to those without BE, but the difference was not statistically significant (*P* = 0.15) (Table 2).

Only hiatal hernia was significantly related with the presence of either Barrett's esophagus or EE (*P* < 0.001). Indeed, hiatal hernia was found in 57.8% of the patients with esophagitis (*n* = 175) compared to 24.5% of those with NEJ (*n* = 106).

With respect to clinical symptoms, heartburn and water brash were the only symptoms correlated significantly with endoscopy-positive GERD (*P* = 0.013 and *P* = 0.005, resp.). There were no significant differences in other clinical findings between the two groups. There was no significant relationship between each LA classification grade and typical reflux symptoms (*P* = 0.129) as well.  Table 3 illustrates the distribution of reflux symptoms in patients with and without erosive changes.

Rapid urease test was positive in a significantly higher number (*n* = 115) of patients with NEJ compared to patients (*n* = 101) with esophagitis (*P* = 0.049). No statistically significant relationship, however, was found between the presence of H. pylori infection and typical reflux symptoms.

Multivariate analysis showed that hiatal hernia (Adjusted OR, 4.56; 95% CI, 3.30–6.31; *P* < 0.001) and H. pylori infection (Adjusted OR, 1.75; 95% CI, 1.24–2.48; *P* < 0.002) were the two significantly related risk factors for esophagitis. Age was the only risk factor significantly related to BE, with BE patients being more likely to be older (Adjusted OR, 1.04; 95% CI, 1.02–1.06; *P* < 0.001) than others.

## 5. Discussion

EE and BE commonly occur in western GERD patients with the approximate prevalence of 30–60% [9] and 5–15% [10] respectively. In Eastern countries, however, lower rates have been reported. For example, in Turkey, the rates of EE and BE in patients with reflux symptoms reported to be about 8%–16% compared to 1.5%, respectively [9]. In the present study, approximately 43% of patients had esophageal lesions, either Barrett's esophagus (4.6%) or erosive esophagitis (38.5%). This shows that the prevalence of these complications in Iranian refluxers is somewhat similar to that seen in western populations. Probably, different risk factors for EE/BE in Iranian patients might be responsible for such different prevalence rates.

Despite the mixed results, different studies have proposed different risk factors for EE. For instance, in Serrano et al. study, a significant relationship was found between severe esophagitis and advanced age, male gender, smoking and H. pylori absence [11]. With regards to GERD symptoms, frequency of heartburn, but not duration or severity of regurgitation, was significantly associated with EE, reported by the Locke et al. [4]. In another study, conducted by Rosaida and Goh, the risk factors for EE were male gender, Indian race, hiatal hernia and alcohol use [12]. Finally, in the Labenz et al. study, male gender, overweight, regular alcohol consumption, history of GERD for more than one year, and being smoker/ex-smoker were the reported risk factors [13].

In accordance with previous studies we found that the presence of hiatal hernia is a strong risk factor for esophagitis (*P* = 0.001) [12, 14]. However, in contrast to some other studies we could not find any significant relationship between hiatal hernia and BE [15].

We found that patients with BE were more likely to be older (*P* = 0.001) than other GERD patients, the finding which is compatible with the previous literature. In fact, BE has been reported to be a rare condition in patients younger that 40–50 years [16]. Although patients with esophagitis had higher mean of age as compared to NEJ patients, our analysis did not show significant difference between the two groups. This finding is opposite to some other studies in which older age has been considered a risk factor for esophagitis [11].

Male gender has also been reported to be an independent risk factor for esophagitis [11–13]. Moreover, different parietal cell mass, lower esophageal function or body mass index between genders have been proposed as possible causes to explain the gender effect [16]. However, in our study, the proportion of male gender was not significantly different between patients who had esophagitis and those who did not. Similarly, we could not find any significant effect for gender to be considered as a risk factor for BE.

The relationship between H. pylori and the development of GERD, EE and BE has not yet been fully understood. However, with regards to the role of H pylori in GERD, it has been suggested that the infection might have some relationships with GERD symptoms. In fact, H pylori gastritis might result in acid hyposecretion and finally loss of burning sensation, while feeling of regurgitation remains intact (volume refluxers) [9]. In the present study, we found that a significantly greater proportion of patients with NEJ, as compared to other patients, had H. pylori infection (*P* = 0.04). However, no statistically significant relationship between the presence of H. pylori infection and the typical reflux symptoms was found. Concerning this, there may be possible cause(s) behind this difference that need to be more investigated.

The main objective of the Los Angeles classification development was to provide a clinically relevant stratification for esophagitis severity. GERD symptoms have been inconsistently correlated with endoscopic findings of EE in different studies, some of which favor such correlation, though not with all reflux symptoms [4], and some argue against it [17, 18]. Our study showed no significant correlation between the reflux symptoms and the different endoscopic grades of EE according to the LA classification. No significant difference was also found between the reflux symptoms of patients with EE versus NEJ.

However, there are some other factors which should be taken into account. For example, we found that 51 out of 88 patients with peptic ulcer did not have any evidence of EE. As it is well known that peptic disease might explain and/or contribute to the development of reflux symptoms such as heartburn or regurgitation, we should accept that peptic disease might be an underlying cause for GERD in a number of our NEJ patients [19]. Similarly, esophageal hypersensitivity should also be considered in which the individual might present with symptoms similar to GERD while having nothing more than a normal esophageal acid exposure [20].

In this study we had some limitations as well. For example, the study was conducted in Tehran region which may not exactly represent the problem in the whole country, although it is the most populated region of Iran. Furthermore, it was difficult for us to obtain accurate data about other important variables such as patients' diet which might have confounded the study results. Hence, conducting similar studies in country-wide scales is warranted to find a better estimation of BE/EE prevalence in the whole Iranian population and their clinically relevant risk factors.

In conclusion, we have shown within a cross-sectional study that the prevalence of GERD complications such as BE and EE in Iranians are close to that seen in developed countries. In addition, H. pylori infection and the presence of hiatal hernia may be strong risk factors for esophagitis in Iran. Similarly, older age could be considered a significant risk factor for the development of BE in GERD. Finally, reflux symptoms seem do not reliably predict simultaneous esophageal lesions on endoscopy.

## Figures and Tables

**Table 1:** Background characteristics of the study groups.

Characteristics	NEJ^†^	EE + BE^†^	*P* value
	(*n* = 433)	(*n* = 303)	
Age (years)	47.97 ± 16.58	50.14 ± 16.9	0.08
Sex			
Male	235 (54.3)	176 (58.1)	0.30
Female	197 (45.4)	127 (41.9)	
BMI (kg/m^2^)	24.52 ± 2.39	24.72 ± 2.31	0.26
Obesity			
Yes	173 (40)	126 (41.6)	0.66
No	257 (59.4)	175 (57.8)	
Asthma			
Yes	14 (3.2)	11 (3.6)	0.77
No	419 (96.8)	292 (96.4)	
Cancer			
Yes	71 (16.4)	38 (12.5)	0.15
No	362 (83.6)	265 (87.5)	
Smoking			
Yes	120 (27.7)	85 (28.1)	0.92
No	313 (72.3)	218 (71.9)	
Alcohol			
Yes	37 (8.5)	29 (9.6)	0.63
No	396 (91.5)	274 (90.4)	
Hiatal			
Yes	106 (24.4)	175 (57.8)	0.001
No	327 (75.5)	128 (42.2)	
RUT			
Yes	115 (26.6)	101 (33.3)	0.04
No	318 (73.4)	202 (66.7)	
PUD			
No	383 (88.5)	265 (87.5)	0.62
Gastric	14 (3.2)	14 (4.6)	
Duodenal	36 (8.3)	24 (7.9)	

^†^Data are mean ± standard deviation or frequency and percent as appropriate.

**Table 2:** Background characteristics of the study groups.

Characteristics	Barrett^†^		*P* value
	Yes	No	
	(*n* = 34)	(*n* = 702)	
Age (years)	59.19 ± 15.24	48.36 ± 16.65	0.001
Sex			
Male	23 (67.6)	388 (55.3)	0.15
Female	11 (32.3)	314 (44.7)	
BMI (kg/m^2^)	23.96 ± 1.92	24.63 ± 2.37	0.01
Obesity			
Yes	11 (32.4)	288 (41)	0.36
No	22 (64.7)	410 (58.4)	
Asthma			
Yes	0	25 (3.6)	0.62
No	34 (100)	677 (96.4)	
Cancer			
Yes	3 (8.8)	106 (15.1)	0.31
No	31 (91.2)	596 (84.9)	
Smoking			
Yes	10 (29.4)	195 (27.8)	0.83
No	24 (70.6)	507 (72.2)	
Alcohol			
Yes	3 (8.8)	63 (9)	1.0
No	31 (91.2)	639 (91)	
Hiatal			
Yes	14 (41.2)	267 (38)	0.50
No	20 (58.8)	435 (62)	
RUT			
Yes	12 (35.3)	204 (29.1)	0.43
No	22 (64.7)	498 (70.9)	
PUD			
No	33 (97.1)	615 (87.6)	0.24
Gastric	0	28 (4)	
Duodenal	1 (2.9)	59 (8.4)	

^†^Data are mean ± standard deviation or frequency and percent as appropriate.

**Table 3:** Comparison of reflux symptoms in study groups.

Symptoms	NEJ^†^	EE + BE^†^	*P* value
	(*n* = 433)	(*n* = 303)	
Heartburn	254 (58.7)	205 (67.7)	0.013
Regurgitation	237 (54.7)	173 (57.1)	0.53
Heartburn + Regurgitation	332 (76.7)	245 (78.4)	0.20
Atypical chest pain	139 (32.1)	103 (34.0)	0.59
Abdominal pain	115 (26.6)	67 (22.1)	0.17
Water brash	38 (8.8)	47 (15.5)	0.005
Nausea/vomiting	63 (14.5)	46 (15.2)	0.81
Anorexia	62 (14.3)	45 (14.9)	0.84
Dysphagia	27 (6.2)	19 (6.3)	0.98
Chronic cough	67 (15.5)	59 (19.5)	0.16
Hoarseness	31 (7.2)	33 (10.9)	0.08
Hiccup	2 (0.5)	2 (0.7)	1.0
Weight loss	55 (12.7)	48 (15.8)	0.23

^†^Data are frequency and percent.

## References

[1] Dent J, El-Serag HB, Wallander MA, Johansson S (2005). Epidemiology of gastro-oesophageal reflux disease: a systematic review. *Gut*.

[2] Nouraie M, Razjouyan H, Assady M, Malekzadeh R, Nasseri-Moghaddam S (2007). Epidemiology of gastroesophageal reflux symptoms in Tehran, Iran: a population-based telephone survey. *Archives of Iranian Medicine*.

[3] Blustein PK, Beck PL, Meddings JB (1998). The utility of endoscopy in the management of patients with gastroesophageal reflux symptoms. *The American Journal of Gastroenterology*.

[4] Locke GR, Zinsmeister AR, Talley NJ (2003). Can symptoms predict endoscopic findings in GERD?. *Gastrointestinal Endoscopy*.

[5] Gerson LB, Shetler K, Triadafilopoulos G (2002). Prevalence of Barrett’s esophagus in asymptomatic individuals. *Gastroenterology*.

[6] Lundell LR, Dent J, Bennett JR (1999). Endoscopic assessment of oesophagitis: clinical and functional correlates and further validation of the Los Angeles classification. *Gut*.

[7] Vakil N, van Zanten SV, Kahrilas P (2006). The Montreal definition and classification of gastroesophageal reflux disease: a global evidence-based consensus. *The American Journal of Gastroenterology*.

[8] Junghard O, Wiklund I (2008). Validation of a four-graded scale for severity of heartburn in patients with symptoms of gastroesophageal reflux disease. *Value in Health*.

[9] Bayrakçi B, Kasap E, Kitapçioğlu G, Bor S (2008). Low prevalence of erosive esophagitis and Barrett esophagus in a tertiary referral center in Turkey. *Turkish Journal of Gastroenterology*.

[10] Yilmaz N, Tuncer K, Tunçyürek M, Ozütemiz O, Bor S (2006). The prevalence of Barrett’s esophagus and erosive esophagitis in a tertiary referral center in Turkey. *Turkish Journal of Gastroenterology*.

[11] Serrano AG, Igea FJG, Jiménez JAL, Hernández CP (2003). Clinical features and endoscopic progression of gastroesophageal reflux disease. *Revista Espanola de Enfermedades Digestivas*.

[12] Rosaida MS, Goh KL (2004). Gastro-oesophageal reflux disease, reflux oesophagitis and non-erosive reflux disease in a multiracial Asian population: a prospective, endoscopy based study. *European Journal of Gastroenterology and Hepatology*.

[13] Labenz J, Jaspersen D, Kulig M (2004). Risk factors for erosive esophagitis: a multivariate analysis based on the proGERD study initiative. *The American Journal of Gastroenterology*.

[14] Jones MP, Sloan SS, Rabine JC, Ebert CC, Huang CF, Kahrilas PJ (2001). Hiatal hernia size is the dominant determinant of esophagitis presence and severity in gastroesophageal refluxdisease. *The American Journal of Gastroenterology*.

[15] Cameron AJ (1999). Barrett’s esophagus: prevalence and size of hiatal hernia. *The American Journal of Gastroenterology*.

[16] Ford AC, Forman D, Reynolds PD, Cooper BT, Moayyedi P (2005). Ethnicity, gender, and socioeconomic status as risk factors for esophagitis and Barrett’s esophagus. *The American Journal of Epidemiology*.

[17] Voutilainen M, Sipponen P, Mecklin J, Juhola M, Färkkilä M (2000). Gastroesophageal reflux disease: prevalence, clinical, endoscopic and histopathological findings in 1128 consecutive patients referred for endoscopy due to dyspeptic and reflux symptoms. *Digestion*.

[18] Okamoto K, Iwakiri R, Mori M (2003). Clinical symptoms in endoscopic reflux esophagitis: evaluation in 8031 adult subjects. *Digestive Diseases and Sciences*.

[19] Barkun A, Leontiadis G (2010). Systematic review of the symptom burden, quality of life impairment and costs associated with peptic ulcer disease. *The American Journal of Medicine*.

[20] Rodriguez-Stanley S, Robinson M, Earnest DL, Greenwood-Van Meerveld B, Miner PB (1999). Esophageal hypersensitivity may be a major cause of heartburn. *The American Journal of Gastroenterology*.

